# A Case of Obstructive Shock and Respiratory Failure Caused by Chest Wall Hematoma

**DOI:** 10.7759/cureus.87942

**Published:** 2025-07-14

**Authors:** Hiroshi Adachi, Kwonil Choi, Motohiro Shimizu

**Affiliations:** 1 Intensive Care Department, Ryokusenkai Yonemori Hospital, Kagoshima, JPN

**Keywords:** chest wall hematoma, compression asphyxia, impaired ventilation, obstructive shock, respiratory failure

## Abstract

A woman in her 70s undergoing dialysis developed respiratory failure and acute circulatory failure accompanied by bleeding after cardiac surgery. A large chest wall hematoma was found in the right anterior chest, and the CT scan showed that the hematoma was compressing the right thoracic cage. After emergency surgery to stop the bleeding and remove the hematoma, the patient’s respiratory and hemodynamic status improved immediately. In this case, the elderly dialysis patient had undergone a sternotomy, which made the thoracic cage more susceptible to external compression and reduced its ability to expand. The rapidly developing hematoma compressing the thoracic cage caused respiratory failure due to ventilation disorders and obstructive shock. This is the first case report in which compression of the thoracic rib cage by a large chest wall hematoma caused respiratory failure and obstructive shock.

## Introduction

Chest wall hematomas can be caused by trauma, anticoagulant use, or tumors and can lead to hemorrhagic or hypovolemic shock [1-3]. The thoracic rib cage is made up of the thoracic vertebrae, ribs, sternum, and intercostal muscles and protects important organs inside the thoracic cavity, such as the heart and lungs. The rib thoracic cage expands and contracts three-dimensionally to change lung volume and is essential for efficient breathing. Restricting the mobility of the rib thoracic cage can affect respiratory function. Strong external forces that continuously compress the thoracic rib cage can cause fatal injuries such as mechanical asphyxia, compression asphyxia, and crowd crush injuries [4-6]. The substantial external pressure directly impedes thoracic expansion and respiratory excursions. Concurrently, an acute elevation in intrathoracic pressure can compromise venous return and consequently diminish cardiac output [4-6]. To the best of our knowledge, this is the first case report in which sustained compression of the thoracic rib cage by a large chest wall hematoma caused respiratory failure due to impaired ventilation and obstructive shock.

## Case presentation

The patient was a woman in her 70s with chronic kidney disease due to focal segmental glomerulosclerosis, who had started heparin-anticoagulated hemodialysis three times a week one month prior and had residual urine output. The patient had no pre-existing coagulation abnormalities (Table 1), with a history of hemiarch repair for Stanford type A aortic dissection and stent-graft placement using the Najuta Thoracic Stent Graft System® (SB-Kawasumi Laboratories, Inc., Kanagawa, Japan), a semi-custom-made fenestrated stent graft system for Stanford type B aortic dissection. The patient underwent the Bentall procedure on cardiopulmonary bypass for severe aortic regurgitation through a median sternotomy, and the sternum was closed with wires. Anticoagulation for cardiopulmonary bypass was achieved using heparin. Inhaled nitric oxide (iNO) therapy was initiated to treat pulmonary hypertension after weaning from cardiopulmonary bypass. The patient exhibited unstable respiratory status and hemodynamics upon ICU admission. However, no signs of overt bleeding, such as subcutaneous, mucosal, or surgical wound bleeding, were noted. The intraoperative fluid balance was +9770 mL, and the patient had significant postoperative weight gain (Table 2).

**Table 1:** Pre- and Post-operative Coagulation Parameters After surgery, blood tests revealed slight coagulation abnormalities. APTT, activated partial thromboplastin time; Fib, fibrinogen quantity; Plt, platelet count; PT-INR, prothrombin time-international normalized ratio

	Pre-scheduled Surgery	Post-scheduled Surgery	Three-Hour Post-emergency Surgery
Plt (×10^4^/μL）	21.6	7.9	5.9
PT-INR	1.05	1.28	1.22
APTT (seconds)	None	42.6	36.8
Fib (mg/dL)	None	162	177

**Table 2:** Pre- and Post-scheduled Surgery Body Weight Change Body weight increased significantly from pre- to post-scheduled surgery.

Pre-operative Dry Weight on Dialysis (kg)	Post-scheduled Surgery Weight (kg)
45.3	58.7

**Figure 1:**
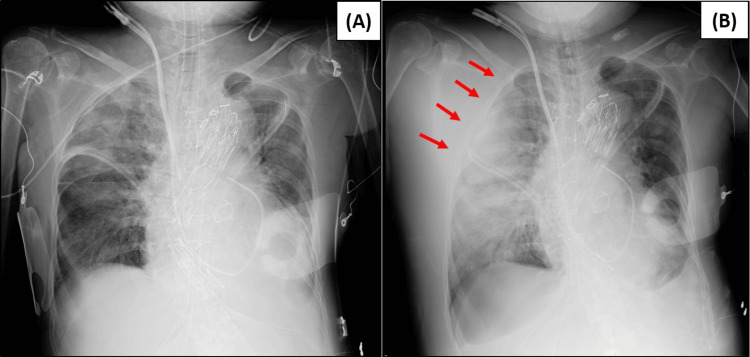
Chest X-rays: Post-scheduled Surgery (Pre-emergency Surgery) Chest X-ray revealed that the right upper thorax was compressed by the large hematoma when compared to the chest X-ray taken immediately after surgery (red arrow). (A) Chest X-ray taken immediately after surgery. (B) Chest X-ray taken after chest wall hematoma was identified.

**Figure 2:**
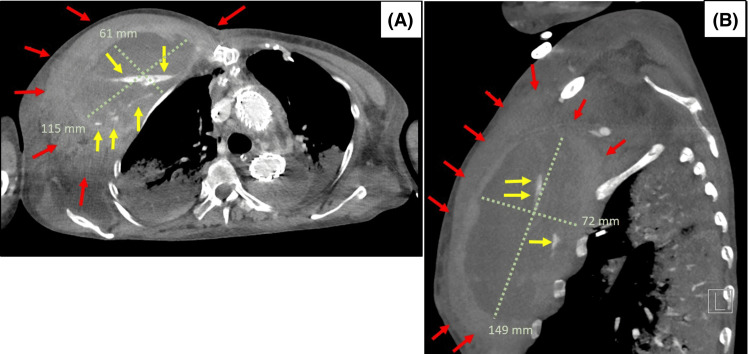
Arterial Phase Chest CT Images: Post-scheduled Surgery (Pre-emergency Surgery) Chest CT images revealed a large hematoma (red arrow) with extravasation (yellow arrow) significantly compressing the right thoracic rib cage. The light green lines indicate the measurements of the chest hematoma. (A) Axial view of CT imaging of chest wall hematoma. (B) Sagittal view of CT imaging of chest wall hematoma.

Shortly after ICU admission, the patient developed hemorrhagic shock. Hemodynamics temporarily improved with fluid infusion and blood transfusion. However, hemodynamics deteriorated again, and respiratory function worsened. Despite adequate blood transfusions, hemodynamic improvement was suboptimal. Four and a half hours post-surgery, significant right anterior chest wall swelling was noted. No obvious bleeding diathesis, such as subcutaneous or mucosal bleeding, was observed in other areas. Chest X-ray taken approximately five hours post-surgery revealed compression of the right upper thorax by a large hematoma, a change from the image taken immediately after surgery (Figure 1). Subsequent CT imaging revealed a large hematoma, with extravasation from a branch of the internal thoracic artery, likely damaged during surgical manipulation, compressing the right thoracic rib cage (Figure 2). Emergency surgery to stop the bleeding and remove the large chest wall hematoma was performed immediately. A transverse incision was made in the right chest wall. Upon incising the fascia, a large amount of accumulated blood was released. After evacuating as much of the hematoma as possible, an actively bleeding artery was identified and ligated with sutures to achieve hemostasis. Oxygenation and hemodynamics improved immediately after the hematoma was removed (Table 3). Subsequently, the partial pressure of oxygen (PaO_2_)/fraction of inspired oxygen (FiO_2_) ratio increased despite reduced ventilator settings (Table 4). Concurrently, the vasopressor dosage was significantly decreased, and the cardiac index showed a significant increase (Table 5).

**Table 3:** Respiratory and Hemodynamic Parameters During Emergency Surgery Immediately after hematoma removal, SpO_2_ and MAP increased and HR decreased compared to at incision, while mPAP remained unchanged and mCVP slightly decreased. FiO_2_, fraction of inspired oxygen; HR, heart rate; MAP, mean arterial pressure; mPAP, mean pulmonary arterial pressure; mCVP, mean central venous pressure; SpO_2_, oxygen saturation

	At Incision	Post-hematoma Evacuation
FiO_2_ (%)	100	100
SpO_2_ (%)	90	95
HR (bpm)	97	80
MAP (mmHg)	72	99
mPAP (mmHg)	31	31
mCVP (mmHg)	21	20

**Table 4:** Respiratory Parameters: Post-scheduled Surgery, Pre- and Post-emergency Surgery Before emergency surgery, the P/F ratio worsened compared to that at ICU admission, despite escalated ventilator settings. However, after emergency surgery, the P/F ratio increased compared to that before emergency surgery, even with de-escalated ventilator settings. FiO_2_, fraction of inspired oxygen; MV, minute volume; P/F ratio, PaO_2_/FiO_2_ ratio; PaCO_2_, partial pressure of carbon dioxide; PaO_2_, partial pressure of arterial oxygen; PEEP, positive end expiratory pressure; PIP, peak inspiratory pressure; VT, tidal volume

	ICU Admission (Post-scheduled Surgery)	Pre-emergency Surgery	Post-emergency Surgery
pH	7.209	7.197	7.316
PaCO_2_ (mmHg)	73.3	63.6	51.3
PaO_2_ (mmHg)	73.4	74.9	93.3
HCO_3_^-^ (mmol/L)	29.2	24.6	26.2
FiO_2_ (%)	60	70	60
P/F ratio	122	107	155.5
PEEP (cmH_2_O）	10	15	10
PIP (cmH_2_O）	26	32	29
VT (mL)	353	432	439
MV (L/minute)	6	6.1	6.2

**Table 5:** Hemodynamic Parameters, Vasopressors, and Hemoglobin Levels High doses of vasopressors were administered at ICU admission. Prior to emergency surgery, mCVP increased and CI decreased compared to the values before ICU admission. Following emergency surgery, the vasopressor dosage was significantly reduced, and the cardiac index significantly increased, compared to before emergency surgery. Before ECUM, vasopressors were further tapered until only small amounts of dopamine and dobutamine were administered CI, cardiac index; ECUM, extracorporeal ultrafiltration method; HR, heart rate; iNO, inhaled nitric oxide; MAP, mean arterial pressure; mCVP, mean central venous pressure; mPAP, mean pulmonary arterial pressure

	Post-scheduled Surgery (at ICU admission)	Pre-emergency Surgery	Post-emergency Surgery	Pre-ECUM (Five-Hour Post-emergency Surgery)
HR (bpm)	97	94	85	73
MAP (mmHg)	82	81	113	106
mPAP (mmHg)	26	28	35	31
mCVP (mmHg)	11	17	18	21
CI (L/minute/m^2^)	3.2	2.2	2.9	2.2
Dopamine (mg/hour)	9	9	3.9	3
Dobutamine (mg/hour)	27	27	15	3
Norepinephrine (mg/hour)	0.2	0.5	0.3	None
Epinephrine (mg/hour)	0.25	0.25	0.1	None
Vasopressin (unit/hour)	none	1.2	0.6	None
iNO (ppm)	20	13	13	13
Hb (g/dL)	9.1	8.4	9.4	10.8

Extracorporeal ultrafiltration method (ECUM) was performed on postoperative day (POD) 1, five hours after emergency surgery, as hemodynamic stability further improved (Table 5), and continuous kidney replacement therapy (CKRT) was performed from POD 2 to POD 9. The patient was weaned from mechanical ventilation on POD 10 and transferred from the ICU to a general ward on POD 13. On POD 72, she was transferred to a local hospital for continued rehabilitation with the goal of eventual discharge home.

## Discussion

Chest wall hematomas can result from trauma, anticoagulant use, or tumors [1-3]. Recently, platelet dysfunction in uremic patients and anticoagulation therapy during hemodialysis have also been identified as contributing factors [7]. Elderly patients, especially those with underlying conditions like renal dysfunction and hypertension, face an increased risk of bleeding due to interacting factors such as uremic coagulopathy, microvascular fragility, and hypertension [7]. While these factors are well-known to cause chest wall hematomas that can lead to hemorrhagic or hypovolemic shock [1-3], this case highlights that the large chest wall hematoma, on a chest wall with diminished structural integrity, carries a serious risk of leading to life-threatening complications such as obstructive shock and respiratory failure.

In this case, the patient presented with respiratory failure and hemodynamic deterioration after the Bentall procedure. Chest contrast CT imaging revealed a large chest wall hematoma with active extravasation from a branch of the internal thoracic artery, possibly injured during the surgical procedure. This rapidly expanding hematoma severely compressed the thoracic rib cage, leading to thoracic movement disorder, pulmonary ventilation disorder, and cardiac diastolic dysfunction, which resulted in acute respiratory failure and obstructive shock. However, immediate hemostasis and removal of the hematoma significantly improved her hemodynamic and respiratory status. After hematoma removal, blood pressure and heart rate improved immediately, but central venous pressure (CVP) did not decrease. This was thought to be due to persistent pulmonary hypertension following cardiopulmonary bypass and fluid overload during and after surgery. When a strong external force is applied to the thoracic cage, its compliance decreases, physically preventing its expansion. This impedes air inflow into the lungs, leading to rapid progression of hypoxemia and hypercapnia, resulting in respiratory failure. The intense compression on the thoracic cage also sharply increases intrathoracic pressure, causing venous return impairment and a decrease in preload, which acutely worsens hemodynamic status. The progression and severity of this respiratory and circulatory failure depend on the real-time condition of the lungs, thoracic cage, and hemodynamics. This case was considered to have a pathology similar to mechanical asphyxia, compression asphyxia, and crowd crush injuries [4-6]. Compression of the chest impairs breathing. Compression force applied in the anteroposterior plane restricts the lungs' ability to expand, impairs breathing, and results in the development of progressive hypoxemia [6]. Compression of the chest also compresses the heart. Severe compression of the chest increases intrathoracic pressure, which reduces venous return to the heart, dramatically reduces cardiac output, and decreases blood pressure. Extreme chest compression can cause a reduction in cardiac output independent of hypoxemia and can occur very rapidly [6]. Early surgical evacuation is a crucial intervention in cases of large hematomas and hemodynamic instability [7], as well as in the presence of respiratory failure.

In elderly individuals, the subcutaneous and soft tissues are characterized by increased fragility and reduced structural integrity. This inherent vulnerability means that when bleeding occurs, such as from trauma or spontaneous events, effective hemostasis is often compromised. This leads to a more extensive and rapid dissemination of hemorrhage, frequently resulting in the formation of significantly larger hematomas.

As individuals age, the thoracic cage undergoes significant changes, including the calcification of costal cartilage, degeneration of intervertebral discs, calcification of joints, and hardening of ligaments, all of which reduce both its compliance and flexibility. Concurrently, decreased bone density, progressive osteoporosis, and deformities of the thoracic vertebrae further compromise the overall stability of the thoracic cage. These structural alterations, combined with the atrophy of respiratory muscles with intercostals and diaphragm, lead to weakened inspiratory strength and reduced lung elastic recoil, making elderly individuals highly susceptible to more severe and rapid respiratory inhibition when subjected to external compression. Consequently, they are prone to experiencing faster and more critical respiratory failure than younger individuals, even when exposed to the same amount of force [8].

Dialysis patients often have reduced bone strength and reduced muscle strength. The thoracic cage, after median sternotomy followed by sternum closure with wires, becomes crushable under external pressure.

In the presented case, the patient's advanced age, dialysis status, and immediately prior median sternotomy, and the weak respiratory muscle strength rendered the thoracic cage vulnerable. Consequently, the sudden onset of a large chest wall hematoma severely compromised its integrity, leading to impaired chest wall expansion. This, in turn, precipitated acute respiratory failure and obstructive shock. For patients with a fragile thoracic cage and diminished respiratory muscle strength, as observed in this case, it is crucial to recognize the potential for chest wall hematomas to cause such severe clinical deterioration, enabling early detection and intervention.

## Conclusions

Chest wall hematomas can be caused by trauma, anticoagulant use, or tumors, and can lead to hemorrhagic or hypovolemic shock. This case highlights the importance of recognizing that a rapidly progressing large chest wall hematoma can cause respiratory failure and obstructive shock, especially in patients whose thoracic cage is easily compressed and whose ability to expand the thoracic cage is weak. It also emphasizes the importance of early detection and, in cases of large hematomas accompanied by hemodynamic instability and respiratory failure, the necessity of performing prompt surgical removal. In conclusion, this report enriches our understanding of respiratory failure and obstructive shock caused by compression of the thoracic cage by a large chest hematoma.
